# Supplementary material for: Early-life stress induces persistent astrocyte dysfunction associated with fear generalisation
Source: eLife. 2025 Feb 5;13:RP99988. doi: 10.7554/eLife.99988 (PMC11798576; doi:10.7554/eLife.99988)
Supplement: Supplementary file 1. [file elife-99988-supp1.docx]

**Supplementary File 1**

**Statistics table**

| **Figure 1** | |
| --- | --- |
| 1B | **CORT at P45**  P=0.01; Naïve=50.1±6.9 ng/ml, N=9; ELS=106.1±17.2 ng/ml, N=11; mean±s.e.m |
| 1D | **Time in periphery Naïve vs ELS**  Two-tailed unpaired t-test, t= 0.7542, P=0.4591;  Naïve=241.8±10.33 seconds, N=9; ELS=230.3±10.27 seconds, N=14; mean±s.e.m |
| 1E | **Time in center Naïve vs ELS**  Two-tailed unpaired t-test, t= 0.7542, P=0.4591;  Naïve= 58.17±10.33 seconds, N=9; ELS= 69.70±10.27 seconds, N=14; mean±s.e.m |
| 1F | **Total distance travelled in OF Naïve vs ELS**  Two-tailed unpaired t-test, t=2.128, P=0.0454;  Naïve= 17.04±2.3 meters, N=9; ELS= 22.82±1.602 meters, N=14; mean±s.e.m |
| 1H | **Time in closed arms Naïve vs ELS**  Two-tailed unpaired t-test, t= 2.34, P= 0.0276;  Naïve= 196.8± 9.763 seconds, N=16; ELS= 231.9± 11.16 seconds, N=11; mean±s.e.m |
| 1I | **Time in open arms Naïve vs ELS**  Two-tailed unpaired t-test, t= 2.34, P= 0.0276;  Naïve= 103.2± 9.763 seconds, N=16; ELS= 68.13± 11.16 seconds, N=11; mean±s.e.m |
| 1J | **Total distance travelled in EPM Naïve vs ELS**  Two-tailed unpaired t-test, t= 2.167, P= 0.0439;  Naïve= 8.167 ± 0.6223 meters, N=9; ELS= 11.03 ± 1.079 meters, N=11; mean±s.e.m |
| **Figure 2** | |
| 2C | **Fear conditioning CS- CS+ Naîve vs ELS**  Two-way ANOVA; effect of group: F (3, 360) = 64.48, P<0.0001;  Tukey’s multiple comparison test,  Naïve CS- vs ELS CS-: P= 0.2124,  Naïve CS+ vs ELS CS+: P= 0.2656,  Naïve CS- vs Naîve CS+: P<0.0001,  ELS CS- vs ELS CS+: P<0.0001,  Naïve CS-= 18.34 ± 3.714 % Freezing, N=15 mice  Naïve CS+= 37.18 ± 8.119 % Freezing, N=15 mice  ELS CS-=13.55±3.491 % Freezing, N=17 mice  ELS CS+=41.67±6.869 %Freezing, N=17 mice  Mean ± s.e.m |
| 2D | **Discrimination test Naîve vs ELS**  Two-way ANOVA; effect of group: F (3, 360) = 28.32, P<0.0001;  Tukey’s multiple comparison test,  Naïve CS- vs ELS CS-: P <0.0001,  Naïve CS+ vs ELS CS+: P= 0.9197,  Naïve CS- vs Naîve CS+: P<0.0001,  ELS CS- vs ELS CS+: P=0.0009,  Naïve CS-= 30.57±1.966% Freezing., N=15 mice  Naïve CS+= 56.22±2.991% Freezing., N=15 mice  ELS CS-=46.03±2.9 % Freezing., N=17 mice  ELS CS+=58.34±2.2 % Freezing., N=17 mice  Mean±s.e.m |
| 2E | **Discrimination index Naîve vs ELS**  Two-way ANOVA; effect of group F (1, 30) = 9.701, P=0.004;  Naïve= 0.3629±0.06059; N=15 mice  ELS=0.1275±0.03533, N=17 mice  mean±s.e.m |
| 2H | **LTP Naîve vs ELS**  Unpaired t-test, P=0.0028;  Naïve=142.1±8.720%, N=15  ELS=106.0±6.058, N=13  mean±s.e.m |
| 2L | **c-Fos density Naîve vs ELS**  Mann-Withney test, P<0.0001, U= 228,  Naïve=17.42±0.9427 c-Fos density/10^-3^mm^3^, n=39 slices from N=10 mice ELS=25.45±1.044 c-Fos density/10^-3^mm^3^, n=37 slices from N=10 mice mean±s.e.m. |
| **Figure 3** | |
| 3C | **Nuclear:cytosolic GR ratio Naîve vs ELS**  P=0.026;  Naïve=0.23±0.06 a.u., N=8  ELS=0.48±0.08 a.u., N=8  mean±s.e.m. |
| 3F | **GFAP**  P=0.02, unpaired t-test;  Naïve=1±0.17 a.u., N=11  ELS=0.5±0.1 a.u, N=12  mean±s.e.m. |
| 3G | **Cx43**  P=0.008, unpaired t-test;  Naïve=1±0.15 a.u., N=11  ELS=0.45±0.12 a.u., N=14  mean±s.e.m. |
| 3H | **GLT-1**  P=0.8, unpaired t-test;  Naïve=1±0.29 a.u., N=6  ELS=0.9±0.21 a.u. N=16  mean±s.e.m |
| **Figure 4** | |
| 4F | Calcium event frequency Naïve vs ELS  Two-tailed unpaired t test, t=4.894, P <0.0001  Naïve= 3.127±0.08503 Hz, n=25 astrocytes from N=8 animals  ELS= 2.444 ±0.1106 Hz, n=25 astrocytes from N=8 animals |
| 4H | Calcium event amplitude Naïve vs ELS  Mann-Whitney test, P=0.0165  Naïve= 0.084±0.006979 au, n=22 astrocytes from N=8 animals  ELS= 0.05818±0.005380 au, n=17 astrocytes from N=8 animals |
| 4J | Calcium event area Naïve vs ELS  Two-tailed unpaired t test, t=5.284, P <0.0001  Naïve= 4.642±0.3611 µm^2^, n=25 astrocytes from N=8 animals  ELS= 2.540±0.1670 µm^2^, n=25 astrocytes from N=8 animals |
| 4L | Calcium event rise time Naïve vs ELS  Two-tailed unpaired t test, t=2.222, P=0.0311  Naïve= 4.782±0.1454 seconds, n=25 astrocytes from N=8 animals  ELS= 5.299 ±0.1821 seconds, n=25 astrocytes from N=8 animals |
| 4N | Calcium event duration Naïve vs ELS  Two-tailed unpaired t test, t=0.7217, P=0.4740  Naïve= 8.987±0.2189 seconds, n=25 astrocytes from N=8 animals  ELS= 9.3075 ±0.3869 seconds, n=25 astrocytes from N=8 animals |
| 4P | Calcium event decay time Naïve vs ELS  Mann-Whitney test, P=0.2164  Naïve= 7.136±0.3719 seconds, n=25 astrocytes from N=8 animals  ELS= 8.147±0.6189 seconds, n=25 astrocytes from N=8 animals |
| **Figure 5** | |
| 5D | **Fear conditioning CS- CS+ eGFP vs CalEx vs dnCx43**  Two-way ANOVA; effect of group: F (5, 463) = 78.39, P<0.0001;  Tukey’s multiple comparison test,  eGFP CS- vs dnCx43 CS-: P=0.1940,  eGFP CS- vs CalEx CS-: P=0.9758,  dnCx43 CS- vs CalEx CS-: P=0.7027,  eGFP CS+ vs dnCx43 CS+: P=0.9536,  eGFP CS+ vs CalEx CS+: P=0.6680,  dnCx43 CS+ vs CalEx CS+: P=0.9761,  eGFP CS-=11.24±1.875 % Freezing., N=12 mice  CalEx CS-=9.182±2.247 % Freezing., N=11 mice  dnCx43 CS-=5.515±1.936 % Freezing., N=17 mice  eGFP CS+= 39.33±6.443 % Freezing., N=12 mice  CalEx CS+=35.22±7.464 % Freezing., N=11 mice  dnCx43 CS+=37.14±6.923 % Freezing., N=17 mice  mean± s.e.m |
| 5E | **Discrimination test eGFP vs CalEx vs dnCx43 freezing responses**  Two-way ANOVA; effect of group:F (5, 457) = 25.29, P<0.0001;  Tukey’s multiple comparison test,  eGFP CS- vs dnCx43 CS-: P=0.0293,  eGFP CS- vs CalEx CS-: P<0.0001,  dnCx43 vs CalEx: P=0.0005,  eGFP CS+ vs dnCx43 CS+: P>0.9999,  eGFP CS+ vs CalEx CS+: P>0.9999,  dnCx43 CS+ vs CalEx CS+: P>0.9999  eGFP CS-= 28.73±2.342 % Freezing., N=12 mice  CalEx CS-= 53.00±1.203 % freezing., N=11 mice  dnCx43 CS-= 38.81±1.957 % Freezing., N=17 mice  eGFP CS+= 57.79±3.314 % Freezing., N=12 mice  CalEx CS+= 57.02±4.219 % Freezing., N=11 mice  dnCx43 CS+= 57.32±4.344 % Freezing., N=17 mice  mean± s.e.m. |
| 5F | **Discrimination index eGFP vs CalEx vs dnCx43**  Two-way ANOVA; effect of group:F (2, 226) = 12.24, P<0.0001;  Tukey’s multiple comparison test,  eGFP vs dnCx43: P= 0.0137,  eGFP vs CalEx: P<0.0001,  dnCx43 vs CalEx: P= 0.0352,  eGFP DI= 0.3636±0.03681;  CalEx DI =0.05023±0.05221;  dnCx43 DI= 0.1989±0.03771; |
| 5G | **LTP eGFP vs CalEx vs dnCx43**  Ordinary one-way ANOVA, group effect: F (2, 31) = 4.221, P=0.0239;  Uncorrected Fisher’s LSD,  eGFP vs CalEx: P=0.0408,  eGFP vs dnCx43: P=0.0207,  CalEx vs dnCx43: P=0.8848,  eGFP=139.0±17.70 % baseline., N=5;  CalEx=100.3±7.485 % baseline., N=9;  dnCx43=98.31±8.635 % baseline., N=16;  mean± s.e.m |
| 4L | **c-Fos density eGFP vs CalEx vs dnCx43**  Ordinary one-way ANOVA, group effect: F (2, 43) = 14.91, P<0.0001;  Tukey’s multiple comparison test,  eGFP vs CalEx: P<0.0001,  eGFP vs dnCx43: P=0.0005,  CalEx vs dnCx43: P=0.8807  eGFP=17.97±0.8195 c-Fos density/10^-3^mm^3.^, n=17 slices from N=5 mice; CalEx=27.66±1.398 c-Fos density/10^-3^mm^3.^, n=17 from N=5 mice; dnCx43=26.65±2.173 c-Fos density/10^-3^mm^3.^, n=12 slices from N=5 mice; mean±s.e.m |
| **Figure supplements** | |
| **Figure 2 – Figure supplement 1** | |
| S1A | **Naïve Auditory Discriminative Fear Conditioning Female vs Male**  **CONDITIONING**  Cs-  Two-way ANOVA; effect of sex: F(1, 13)=1.20; P=0.291  Naïve Male=16.31±3.33; N=8  Naïve Female=23.9±5.12; N=7  Cs+/US  Two-Way ANOVA; effect of sex: F(1, 13)=0.33; P=0.574  Naïve Male=36.11±8.54; N=8  Naïve Female=39.49±8.08; N=7 |
| S1B | **Naïve Auditory Discriminative Fear Conditioning Female vs Male**  **TESTING**  Cs-  Two-way ANOVA; effect of sex: F (1, 13) = 0.5316; P=0.4789  Naïve Male=27.97±2.18; N=8  Naïve Female=36.79±1.241; N=7  Cs+/US  Two-Way ANOVA; effect of sex: F (1, 13) = 0.1696; P=0.6872  Naïve Male=54.75±2.58; N=8  Naïve Female=58.83±4.84; N=7 |
| S1C | **Naïve Auditory Discriminative Fear Conditioning Female vs Male**  **Discrimination Index**  Two-Way ANOVA; effect of sex: F(1,13)=0.03; P=0.85  Naïve Male=0.32±0.16; N=8  Naïve Female=0.33±0.06; N=7 |
| S1D | **ELS Auditory Discriminative Fear Conditioning Female vs Male**  **CONDITIONING**  Cs-  Two-way ANOVA; effect of sex: F (1, 15) = 2.109, P=0.167  ELS Male=17.69±4.205; N=8  ELs Female=9.88±3; N=9  Cs+/US  Two-Way ANOVA; effect of sex: F (1, 15) = 9.638, P=0.0072  ELS Male=33.74±6.28; N=8  ELS Female=48.71±7.81; N=9 |
| S1E | **ELS Auditory Discriminative Fear Conditioning Female vs Male**  **TESTING**  Cs-  Two-way ANOVA; effect of sex: F (1, 15) = 1.484, P=0.2419  ELS Male=42.17±2.997; N=8  ELs Female=50.39±4.011; N=9  Cs+  Two-Way ANOVA; effect of sex: F (1, 15) = 1.763, P=0.2041  ELS Male=54.60±2.054; N=8  ELS Female=62.54±3.816; N=9 |
| S1F | **ELS Auditory Discriminative Fear Conditioning Female vs Male**  **Discrimination Index**  Two-Way ANOVA; effect of sex: F (1, 15) = 0.4431; P=0.515  Naïve Male=0.123±0.053; N=8  Naïve Female=0.149±0.033; N=9 |
| **Figure 2 - Figure supplement 2** | |
| S2A | **Auditory Discriminative Fear Conditioning ELS vs. Naïve**  **CONDITIONING**  Two-Way ANOVA; effect of group : F (1,30)=2.613; P=0.116; effect of tone type : F (1, 30) = 53.36, P<0.0001  CS-, Naive vs ELS: P= 0.2543  CS+, Naive vs Els : P= 0.2851  Naive, CS+ vs CS- : P<0.0001  ELS, CS+ vs CS- = P<0.0001  Naive CS-=18.34±3.71; N=15  Naïve CS+/US = 37.18±8.11; N=15  ELS CS- = 13.55±3.49; N=17  ELS CS+/US = 41.67±6.86; N=17 |
| S2B | **Auditory Discriminative Fear Testing ELS vs. Naïve**  **TESTING**  Two-Way ANOVA; effect of group: F (1,28)=0.0014; P=0.9701; effect of tone type : F (1, 32) = 43.23, P<0.0001  CS-, Naive vs ELS: P=0.0128  CS+, Naive vs Els : P=0.726  Naive, CS+ vs CS- : P=0.0001  ELS, CS+ vs CS- = P=0.0016  Naive CS-=30.57±1.96; N=15  Naïve CS+/US =56.22±2.99; N=15  ELS CS- = 46.03±2.92; N=17  ELS CS+/US = 58.34±2.26; N=17 |
| S2F | **c-Fos expression Females vs Males Naive**  Two-Tailed T-Test : T=1.677; P=0.102,  Females=19.74±1.754, N=12  Males= 16.39±1.08, N=27 |
| S2G | **c-Fos expression Females vs Males ELS**  Two-Tailed T-Test : T=2.153; P=0.038,  Females=23.25±1.38, N=18  Males= 27.54±1.429, N=19 |
| **Figure 3 – Figure supplement 1** | |
| S3A | **Normalized GFAP fluorescence Naïve ELS Male Female**  Two-Way ANOVA, effect of group F (1, 29) = 8.915, P=0.0057 ; effect of sex F (1, 29) = 0.04720, P=0.8295  Tukey’s HSD:  Male, Naïve vs ELS: P=0.0.0582  Female, Naïve vs ELS; P=0.0323  Naïve, Male vs Female: P>0.999  ELS, Male vs Female: P=0.7501  Female Naïve=1.00±0.2849, N=8  Male Naïve=1.00±0.2290, N=7  Female ELS=0.4052±0.0762, N=8  Male ELS=0.4859±0.1076, N=10 |
| S3B | **Cx43 fluorescence Naïve ELS Male Female**  Two-Way ANOVA, effect of group F (1, 28) = 11.66, P=0.0020; effect of sex F (1, 28) = 0.005276, P=0.9426  Male, Naïve vs ELS: P=0.0253  Female, Naïve vs ELS; P=0.0201  Naïve, Male vs Female: P>0.999  ELS, Male vs Female: P=0.9189  Female Naïve=1.00±0.09592, N=8  Male Naïve=1.00±0.2407, N=7  Female ELS=0.4452±0.0752, N=8  Male ELS=0.4683±0.1767, N=10 |
| **Figure 4 – Figure supplement 1** | |
| S4A | **Amplitude 2P Naïve ELS Male Female**  Two-Way ANOVA; effect of group F (1, 35) = 9.437; P=0.0041; effect of sex F (1, 35) = 1.904, P=0.1764  Male, Naïve vs ELS: P=0.5478  Female, Naïve vs ELS; P=0.0058  Naïve, Male vs Female: P=0.0425  ELS, Male vs Female: P=0.9627  Female Naïve=0.09±0.012, N=10  Male Naïve=0.071±0.005, N=12  Female ELS=0.0562±0.0111, N=7  Male ELS=0.0595±0.00539, N=10 |
| S4D | **Event Area 2P Naïve ELS Male Female**  Two-Way ANOVA; effect of group F (1, 46) = 41.50; P<0.0001; effect of sex F (1, 46) = 8.116, P=0.0065  Male, Naïve vs ELS: P=0.0385  Female, Naïve vs ELS; P<0.0001  Naïve, Male vs Female: P=0.0002  ELS, Male vs Female: P=0.9702  Female Naïve=5.806±0.562, N=11  Male Naïve=3.721±0.304, N=14  Female ELS=2.484±0.267, N=12  Male ELS=2.592±0.2151, N=13 |
| S4G | **Rise Time 2P Naïve ELS Male Female**  Two-Way ANOVA; effect of group F (1, 46) = 9.458; P<0.0035; effect of sex F (1, 46) = 1.152, P=0.2886  Tukey’s HSD:  Male, Naïve vs ELS: P=0.7971  Female, Naïve vs ELS; P<0.0001  Naïve, Male vs Female: P=0.0021  ELS, Male vs Female: P=0.1002  Female Naïve=4.192±0.1243, N=11  Male Naïve=5.195±0.1388, N=14  Female ELS=5.594±0.1575, N=12  Male ELS=5.027±0.3061, N=13 |
| S4J | **Frequency 2P Naïve ELS Male Female**  Two-Way ANOVA; effect of group F (1, 46) = 22.84; P<0.0001; effect of sex F (1, 46) = 0.01429, P=0.9054  Male, Naïve vs ELS: P<0.0001  Female, Naïve vs ELS; P=0.0419  Naïve, Male vs Female: P=0.2053  ELS, Male vs Female: P0.2671  Female Naïve=2.985±0.132, N=11  Male Naïve=3.288±0.108, N=14  Female ELS=2.558±0.129, N=12  Male ELS=2.339±0.175, N=13 |
| S4M | **Duration 2P Naïve ELS Male Female**  Two-Way ANOVA; effect of group F (1, 46) = 0.7290; P<0.3976; effect of sex F (1, 46) = 0.2611, P=0.6118  Male, Naïve vs ELS: P=0.8877  Female, Naïve vs ELS; P=0.6991  Naïve, Male vs Female: P=0.8304  ELS, Male vs Female: P=0.9806  Female Naïve=8.741±0.3951, N=11  Male Naïve=9.093±0.2480, N=14  Female ELS=9.249±0.5234, N=12  Male ELS=9.362±0.5861, N=13 |
| S4P | **Decay Time 2P Naïve ELS Male Female**  Two-Way ANOVA; effect of group F (1, 46) = 2.459; P<0.1237; effect of sex F (1, 46) = 0.08601, P=0.7706  Tukey’s HSD:  Male, Naïve vs ELS: P=0.9997  Female, Naïve vs ELS; P<0.0718  Naïve, Male vs Female: P=0.5942  ELS, Male vs Female: P=0.3383  Female Naïve=6.570±0.5769, N=11  Male Naïve=7.512±0.4867, N=14  Female ELS=8.858±0.9071, N=12  Male ELS=7.492±0.8382, N=13 |
| **Figure 5 – Figure supplement 2** | |
| S6F | **Event frequency CalEx Male vs Female**  Mann-Whitney U test, U=2, P=0.0087  CalEx- = 2.571±0.4405, N=6  CalEx+ 1.264±0.1027, N=6 |
| S6G | **Amplitude CalEx Male vs Female**  Mann-Whitney U test, U=2, P=0.0411  CalEx- = 1.432±0.04066, N=6  Calex+ =0.0705±0.0063, N=6 |
| S6H | **Event area CalEx Male vs Female**  Mann-Whitney U test, U=6, P=0.0649  CalEx- = 4.428±1.275, N=6  CalEx+ = 2.071±0.2492, N=6 |
| S6I | **Duration CalEx Male vs Female**  Mann-Whitney U test, U=9, P=0.1797  CalEx- = 6.295±0.4358, N=6  CalEx+ = 5.480±0.5079, N=6 |
| S6J | **Rise Time CalEx Male vs Female**  Mann-Whitney U test, U=11, P=0.3095  CalEx- = 5.066±1.060, N=6  CalEx+ = 3.656±0.3663, N=6 |
| S6K | **Decay Time CalEx Male vs Female**  Mann-Whitney U test, U=14, P=0.5887  CalEx- = 5.136±0.5837, N=6  CalEx+ = 4.723±0.5591, N=6 |
| **Figure 5 – Figure supplement 3** | |
| S7A | **EPM – Time in closed arms eGFP CalEx dnCx43**  Kruskal-Wallis Test H=1.465, P=0.4807  eGFP=225.1±15.15, N=7  CalEx=237.9±5.899, N=9  dnCx43=242.9±9.076, N=9 |
| S7B | **EPM – Time in open arms eGFP CalEx dnCx43**  Kruskal-Wallis Test H=1.465, P=0.4807  eGFP=74.88±15.15, N=7  CalEx=62.14±5.899, N=9  dnCx43=57.06±9.076, N=9 |
| S7C | **EPM – Distance Traveled eGFP CalEx dnCx43**  One-Way ANOVA, main effect of condition F (DFn, DFd); P=0.3231  eGFP=11.38±1.379, N=7  CalEx=9.421±0.7355, N=9  dnCx43=9.898±0.6352, N=9 |
| S7D | **Open Field – Time in Periphery eGFP CalEx dnCx43**  Kruskal-Wallis Test H=1.528, P=0.4658  eGFP=36.95±8.727, N=6  CalEx=50.05±8.165, N=11  dnCx43=37.73±6.460, N=12 |
| S7E | **Open Field – Time in Center eGFP CalEx dnCx43**  Kruskal-Wallis Test H=1.528, P=0.4658  eGFP=263.1±8.727, N=6  CalEx=249.9±8.165, N=11  dnCx43=262.3±6.460, N=12 |
| S7F | **Open Field – Distance Traveled eGFP CalEx dnCx43**  Kruskal-Wallis Test H=4.282, P=0.1176  eGFP=22.12±5.799, N=6  CalEx=15.68±1.292, N=11  dnCx43=17.42±1.666, N=12 |
| **Figure 5 – Figure supplement 4** | |
| S8A | **Auditory Discriminative Fear Conditioning eGFP vs CalEx vs dnCx43**  **CONDITIONING**  Two-Way ANOVA; effect of group : F (2, 74) = 1.406; P=0.2516; effect of tone type : F (1, 74) = 159.1, P<0.0001  eGFP, CS- vs CS+/US, P<0.0001  CalEx, CS- vs CS+/US, P<0.0001  dnCx43, CS- vs CS+/US, P<0.0001  CS-, eGFP vs CalEx, P=0.944  CS-, eGFP vs dnCx43, P=0.3372  CS-, dnCx43 vs CalEx, P=0.7121  CS+/US, eGFP vs CalEx, P=0.6879  CS+/US, eGFP vs dnCx43, P=0.8030  CS+/US, dnCx43 vs CalEx, P=0.9891  CS- eGFP = 11.24±2.754, N=12  CS- CalEx = 53±5.859, N=11  CS- dnCx43 = 38.59±3.374, N=17  CS+/US eGFP = 39.33±4.552, N=12  CS+/US CalEx = 35.22±3.492, N=11  CS+/US dnCx43 = 36.3±2.07, N=17 |
| S8B | **Auditory Discriminative Fear Conditioning dGFP vs CalEx vs dnCx43**  **TESTING**  Two-Way ANOVA; effect of group : F (2, 74) = 3.612; P=0.0318; effect of tone type : F (1, 74) = 23.83, P<0.0001  eGFP, CS- vs CS+, P<0.0001  CalEx, CS- vs CS+, P=0.534  dnCx43, CS- vs CS+, P=0.0014  CS-, eGFP vs CalEx, P=0.0007  CS-, eGFP vs dnCx43, P=0.0.2383  CS-, dnCx43 vs CalEx, P=0.0467  CS+/US, eGFP vs CalEx, P=0.9991  CS+/US, eGFP vs dnCx43, P=0.9779  CS+/US, dnCx43 vs CalEx, P=0.9949  CS- eGFP = 28.73±3.826, N=12  CS- CalEx = 53.0±5.859, N=11  CS- dnCx43 = 38.59±3.374, N=17  CS+/US eGFP = 57.79±4.558, N=12  CS+/US CalEx = 57.02±4.644, N=11  CS+/US dnCx43 = 55.74±3.260, N=17 |
| S8C | **Auditory Discriminative Fear Conditioning – Discrimination Index CalEx Male vs Female**  Two-Way ANOVA, main effect of sex F (5, 54) = 3.726; P=0.0057 |
| S8D | **Auditory Discriminative Fear Conditioning – Discrimination Index dnCx43 Male vs Female**  Two-Way ANOVA, main effect of sex F (5, 90) = 1.435; P=0.2195 |
| **Figure 5 – Figure supplement 5** | |
| S9D | **c-Fos density, eGFP Male vs Female**  Mann-Whitney test, U=17, P=0.9372  Female = 18.16±1.599, N=6  Male = 18.50±1.762, N=6 |
| S9E | **c-Fos density, CalEx Male vs Female**  Mann-Whitney test, U=23, P=0.3502  Female = 28.51±1.982, N=11  Male = 26.10±1.601, N=6 |
| S9F | **c-Fos density, dnCx43 Male vs Female**  Mann-Whitney test, U=1, P=0.0051  Female = 33.32±2.722, N=5  Male = 21.88±1.488, N=7 |
